# A systematic review of the research evidence on cross-country features of illegal abortions

**DOI:** 10.15171/hpp.2017.22

**Published:** 2017-06-14

**Authors:** Farideh Aghaei, Abdolreza Shaghaghi, Parvin Sarbakhsh

**Affiliations:** ^1^Health Education & Promotion Department, Tabriz University of Medical Sciences, Tabriz, Iran; ^2^Medical Education Research Centre, Tabriz University of Medical Sciences, Tabriz, Iran; ^3^Department of Biostatistics and Epidemiology, Tabriz University of Medical Sciences, Tabriz, Iran

**Keywords:** Induced abortion, Criminal abortion, Illegal abortion, Embryotomy

## Abstract

**Background:** There are contrasting debates about abortions and prohibitory regulations posed serious public health challenges especially in underdeveloped and developing countries. Due to paucity of the empirical evidences this study was conducted to explore the existent cumulative knowledge with special focus on the applied methodology.

**Methods:** A comprehensive review of published articles from January 1995 to December 2015 was performed. Several databases including: Embase, PubMed, Cochrane and also databasesof the Iranian medical journals were searched using combinations of relevant Medical Subject Headings (MeSH terms) and their equivalents, i.e., induced abortion, embryotomy, criminal abortion and illegal abortion. The STrengthening the Reporting of OBservational studies in Epidemiology (STROBE) statement for appraisal of the cross-sectional studies and Consolidated Criteria for Reporting Qualitative Research (COREQ) checklist for the qualitative reports were utilized. After removal of duplicates and irrelevant publications 36 articles remained for data analysis.

**Results: ** A wide heterogeneity was observed in the utilized methodology with no standard data collection tool. Face to face interview and self-administered questionnaire were the most common reported data collection/tool respectively. Married and unemployed women of 26-30 years old age group with low socioeconomic backgrounds were the most typical illegal abortees in the included studies.

**Conclusion:** Despite limitation in accessing all relevant publications and including only those reports written in English or Persian languages, the accumulated knowledge might be applicable to develop a potentially inclusive data collection tool and hence, improve the quality of data collection and/or application of a more robust study design in future investigations.

## Introduction

There are contrasting debates about abortions irrespective of the reasons or circumstances in which they were performed. A wide diversity exists in the abortion law and regulation across the globe e.g., it is restrictively illegal in some countries or legal in other countries only when a woman’s life is endangered by the continuation of her pregnancy or other medical reasons. Prohibitory laws and regulation; however, posed serious public health challenges in different countries especially in underdeveloped and developing countries.^1-4^ Induced abortion by definition is intentional termination of a pregnancy by medical or surgical means before the fetus can be viable.^2^ Unsafe abortion; however, refers to ending of a pregnancy by individuals who lack the required medical skills to perform the procedure, its administration in a sub-optimal environment condition which is deficient in the basic and minimal medical standards, or both.^3^ In countries where a total ban has been imposed on induced abortion or it is merely legally allowed under certain conditions many women in consequence; search for clandestine abortion or what literally is called backyard abortion, that is too often unsafe and endanger women’s life or leave serious complications.^4^

It is reckoned that about 13% of maternal death can be attributable to unsafe abortions worldwide and thus considering almost 22 million abortions that are carrying out unsafely each year, 47 000 women die and further 5 million become disabled annually.^5^ Incomplete abortion, post abortion sepsis, hemorrhage, genital injury and abortion related deaths are among the recognized consequences of unsafe abortions. It is predicted that only in developing countries about 5 million women are admitted to hospitals due to complications of unsafe abortion each year and millions of them endure long-term health consequences including infertility and thousands die after an unsafe abortion.^6^

Varying strategies and methodologies have been applied in different studies on the incidence of unsafe abortions, environmental circumstances in which they were performed or on its contributing factors.^4,7-9^ Question about incident(s) of unsafe abortion based on the social networks of abortees^7^ and use of self-administered questionnaire^8^ or interview^9^ as data collection approach, tool or procedure were among the reported applied methodologies in the literature.

Number of conducted studies in Iran on abortion which is only endorsed in cases of life endangerment, rape or severe fetal anomalies is meager. Due to paucity of the empirical evidences both in national and international level about the illegal abortions this study was conducted to explore existent cumulative knowledge on the phenomenon with special focus on the features of conducted studies and applied methodologies to inform future investigations.

## Materials and Methods

A comprehensive review of published articles in international and national scope from January 1995 to December 2015 was performed to appraise research evidence on the applied methodology in the studies of illegal and unsafe abortion. Several electronic databases including: Embase, PubMed, Cochrane, Scopus, Web of Knowledge (ISI), Google Scholar, Global Health, Medline, Proquest, Science Direct and also databases of the Iranian medical journals, i.e., Irandoc, Iranmedex, SID and Magiran were searched.

### Inclusion Criteria

*Types of studies*

This systematic review involved all quantitative and qualitative non-interventional publications published in English and Persian language from January 1995 to December 2015 that recruited women who themselves or their close relatives or friends underwent medical or surgical illegal abortions at any age. The chosen time span was decided to warrant up datedness and propensity of the study findings.

*Types of outcome variables*

Considered primary outcome variables were applied data collection tools and strategies to study illegal abortion. Characteristics of the women who reported to have illegal abortion, attributes of the illegal abortion providers, reasons to seek for induced abortion and conditions in which the abortions had been carried out also incorporated.

### Search strategy

Combinations of Medical Subject Headings (MeSH terms) and their equivalents, i.e., induced abortion**,** abortion rate, embryotomy, criminal abortion and illegal abortion were used to search for relevant scientific evidence (e.g., [illegal abortion [Title/Abstract]) OR criminal abortion [Title/Abstract]) OR Induced abortion [Title/Abstract]) OR embryotomy [Title/Abstract] Filters: Journal Article; Meta-Analysis; Multicenter Study; Observational Study; Published Erratum; Review; Systematic Reviews; Full text; published in the last 10 years; Humans] string was used to search PubMed).

### Selection of studies and data extraction

Two reviewers (FA and AS) independently assessed the eligible studies based on a uniform set of priori quality criteria and all discrepancies in the assessment results were resolved by consensus. A generic data extraction template was constructed to obtain the required data about the pre-determined properties of the included publications.

## Results

**Figure 1:**
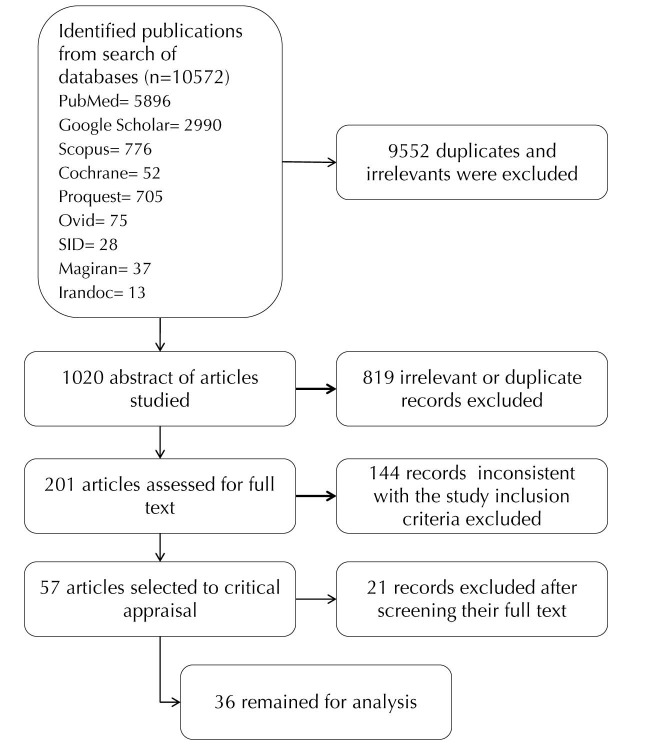
Flow diagram of the included studies in the systematic review of the research evidence on cross-country features of illegal abortions‏.

The primary study search yielded 10 572 articles and after removal of duplicates and irrelevant publications 1020 articles remained for further scrutiny. In the next step, title and abstracts of the articles were investigated to retrieve those publications that fulfill the study objectives. Thus; full text of the 201 articles that considered to have the inclusion criteria were obtained and carefully inspected. Each publication at this stage was assessed based on its quality and strength. To minimize probability of selection bias the STROBE (STrengthening the Reporting of OBservational studies in Epidemiology) statement^10^ for assessment of the cross-sectional studies and COREQ (Consolidated Criteria for Reporting Qualitative Research) checklist^11^ for appraisal of the qualitative study reports were utilized. All disagreements about the quality and eligibility of the identified publications were resolved by consensus and finally 36 articles remained for data analysis (Figure 1).

The extracted data from the identified relevant studies based on the researchers’ names, study type, sample and location were tabulated in Table 1.

A validated data collection instrument was not identified to be applied in studies on illegal abortion. However, different data collection methods including face to face interview, filling of a self-administered questionnaire, in-depth interview, telephone interview and focus group discussion were suggested in the literature for data collection purposes (Table 2).

Other studied features of abortees in the retrieved publications included age, marital status, numbers of children, educational level, employment and socioeconomic status (Table 3).

Extricated data about the reported providers of illegal abortion in the identified publications were summarized in Table 4. As indicated non-skilled individuals were the most reported provider of illegal abortion in the included studies.

The reasons stated by the abortees for requesting an illegal abortion in the included studies were presented in Table 5. Having an unplanned/unwanted pregnancy was the most frequent declared rationale to illegally terminate pregnancy.

Reported places that had been used to perform illegal abortions in the identified studies were displayed in Table 6. Based on the summarized data the frequency of studies that reported performing of abortion cases in unhealthy and improper places (private house or office) is comparable to performing the procedure in healthy and reliable settings (hospitals).

## Discussion

Main purpose of this study was to accumulate the existent scientific evidence about methodological features of empirical studies on illegal abortion. The prime focus; however, was on the data collection tools and methods. A wide heterogeneity was observed in the utilized methodology with no standard data collection tool that was validated for research purposes. Face to face interview^36-41,44^ and application of a self-administered questionnaire^31-33,35,43^ in queries about illegal abortion were the most common reported data collection method respectively. The study’s findings have also revealed that married^25-27,31,33,35,38,39,41,43^ and unemployed women^25-27,29,35,37,38,40,43,44^ of 26-30 years old age group^35-38,41,42^ with 1-2 children^9,13,16,19,25,31,34,35,37,41,43^ and low socioeconomic backgrounds^7,13,16,17,21,27,38,39,40,42^ were the most typical illegal abortion seekers in the included studies. The observed partial inconsistency in the attributes of the abortees in the quoted studies; however, may reflect inherent cultural differences regarding pre-marital sexual relationship, out of wedding pregnancies or aberrant methodologies used.

A sizable number of included studies have reported that illegal abortions had been performed by an unskilled person^12,13,25,31,37,41^in unhealthy non-standard or suboptimal conditions.^20,25,28,34,37^ Having desired number of children was the most referred rationale^35-37,41,43^ to seek for illegal termination of a pregnancy in communities where abortion laws for mothers is criminalized.

In general; liberal abortion related laws and regulations may justify the sparse number of studies that were reported to examine illegal abortion in the developed countries^17,18,21,31^ but this may pose restriction in the applicability of the research evidence originated mostly from less developed or developing countries to design research in other countries of the world.

Limitation in accessing all relevant publications and including only those reports written in English or Persian languages were potential sources of bias in this study. In contexts where abortion cases due to prohibitory laws are executed underground, study respondents might be reluctant to give explicit answers regarding their or their relatives and friends experiences on abortion. Therefore, due to all above mentioned reasons interpretation of the findings must be conservative and tempered by the limitation of the imprecise data.

## Conclusion

Conducting research on illegal abortion is challenging specially due to its stigmatized nature and its surrounded prohibitory laws and regulations that might prevent active participations of target populations. To the best of our knowledge this study was the first systematic investigation of research evidence on characteristics of illegal abortees and methodologies that were used to examine illegal abortions.

No gold standard method was identified to pinpoint for recommendation in future studies. However, the existent evidence might be applicable to develop a potentially inclusive data collection tool and hence improve the quality of data collection and/or application of a more robust study design in future investigations.

Use of innovative data collection instruments or methods may potentially surmount challenges in conducting research on this subterranean and criminalized phenomenon in many countries of the world.

## Ethical approval

The study was granted approval from the Medical Ethics Committee of the Tabriz University of Medical Sciences (approval No. TBZMED.REC. 1393.198).

## Competing interests

There are no competing interests.

## Authors’ contributions

FA contributed to the conceptualization and study design, data collection and interpretation, manuscript drafting and its editing. AS’s major role was conceptualization and study design, help in interpretation of the data and critically revising several drafts of the article for improvement of its intellectual content. PS helped greatly in conceptualization and design of the study, data analysis and interpretation and also preparation of the final draft of the article. All authors have read and approved the submitted and revised final version of the manuscript and confirm that no part of this paper is copied from other sources.

## Disclaimer

The authors claim that no part of this paper is copied from other sources.

## Acknowledgments

The authors would like to acknowledge the authors of the published articles and their guidance to design and perform research on illegal abortions that may help to save lives of innocent mothers and unborn babies. This study was financed by a grant from the Research & Technology Vice-Chancellor Office of the Tabriz University of Medical Sciences, Iran.

**Table 1:** Attributes of the included studies in the systematic review of the research evidence on cross-country features of illegal abortions

**Author/ Date**	**Location**	**Study Type**	**Population**	**Sample size**
Koster-Oyekan^12^ (1998)	Zambia	Cross-sectional	1) School girls, 2) Women	1273, 803
Ahmed et al^13^ (1999)	Bangladesh	Qualitative	Women seeking abortion-related care	143
Rasch et al^14^ (2000)	Tanzania	Cross-sectional	Patients with the diagnosis of incomplete abortion	603
Uygur et al^15^ (2000)	Turkey	Cross-sectional	Women who requested abortion	588
Mogilevkina et al^16^ (2000)	Ukraine	Case-control	Women of fertile age (15–49)	1694
Rasch et al^17^ (2002)	Denmark	Case-control	Pregnant women	809
Larsson et al^18^ (2002)	Sweden	Cross-sectional	Women requesting an early pregnancy termination	591
Ban et al^19^ (2002)	Sri Lanka	Cross-sectional	Clients at an abortion clinic	356
Ganatra and Hirve^20^ (2002)	India	Qualitative	1) Married women who had an induced abortion 2) Abortion services’ providers	1717, 159
Ilboudo et al^8^ (2014)	Burkina Faso	Cross-sectional	Women seeking post abortion care	549
Sihvo et al^21^ (2003)	France	Cross-sectional	18 to 44 year old women	1034
Perera et al^22^ (2004)	Sri Lanka	Cross-sectional	Pregnant women	210
Bozkurt et al^23^ (2004)	Turkey	Cross-sectional	Ever married women	1491
Senbeto et al^24^ (2005)	Ethiopia	Cross-sectional	Women aged 15 to 49	1346
Adanu et al^25^ (2005)	Ghana	Cross-sectional	Cases of complicated abortions	150
Osur et al^7^ (2015)	Kenya	Mixed-method	Women treated for complication of unsafe abortion	963
Nojomi et al^26^ (2006)	Iran	Cross-sectional	Women aged 15 to 55 years	2470
Lara et al^27^ (2006)	Mexico	Cross-sectional	Women ages 15 to 55	1792
Maral et al^28^(2007)	Turkey	Cross-sectional	Women aged 15 years or older	2455
Dahlbäck et al^29^ (2007)	Zambia	Cross-sectional	Girls aged 13 to 19 years	34
Hess et al^30^ (2007)	Africa	Qualitative	Women with a history of induced abortion	5
Tsakiridu et al^31^ (2008)	Spain	Cross-sectional	Women prostitutes	212
Rahim and Ara^32^ (2008)	Pakistan	Cross-sectional	Married women in reproductive age	50
Dibaiee and Saadati^33^ (2008)	Iran	Cross-sectional	Women undergone abortion	85
Rasch et al^34^ (2009)	Tanzania	Cross-sectional	Women admitted with alleged miscarriage	751
Erfani^9^ (2011)	Iran	Cross-sectional	Married women aged 15–49	2934
Motavalli et al^35^ (2012)	Iran	Cross-sectional	Married women aged 15–49	1200
Veisi and Zangene^36^ (2012)	Iran	Cross-sectional	Women with a history of induced abortion	91
Ranji^37^ (2012)	Iran	Cross-sectional	Women aged 15 to 45	3250
Nur^38^(2012)	Turkey	Cross-sectional	Ever-married women aged 15-49 years	1264
Souza et al^39^ (2014)	Brazil	Cross-sectional	Women of childbearing age	860
Fusco et al^40^ (2012)	Brazil	Cross-sectional	Women 15-54 years	375
Rocca et al^41^ (2013)	Nepal	Cross-sectional	Women admitted for post abortion care	527
Motaghi et al^42^ (2013)	Iran	Qualitative	Women with a history of abortion / unwanted pregnancy/ service providers	72
Awoyemi and Novignon^43^ (2014)	Nigeria	Cross-sectional	Women between 19–49 years	308
Klutsey and Ankomah^44^ (2014)	Ghana	Case-control	Case: women who had induced abortion Control: never had an induced abortion	380

**Table 2:** Applied data collection methods in the included studies within the systematic review of the research evidence on cross-country features of illegal abortions

**Data collection methods**	**Number of reporting studies**
Face to face -interview	24 (7, 9, 12 ,13, 14, 15, 19, 22, 23, 25, 26, 27,28, 29, 34, 36, 37, 38, 39, 40, 41, 44)
Self-administered questionnaire	13 (8, 12, 16, 17, 18, 24, 31, 32, 33, 35, 43)
In-depth interview	3 (20, 30, 42)
Telephone interview	1 (21)
Focus group discussion	1 (12)

**Table 3:** Characteristics of the illegal abortees in the included studies within the systematic review of the research evidence on cross-country features of illegal abortions

**Characteristics**	**No. of reporting publications**
Mean age	
≤19	5 (14, 24, 29, 34, 35)
20-25	6 (8, 17, 33, 34, 35, 44)
26-30	18 (13, 15, 16, 18, 21, 22, 23, 25, 26, 27, 28, 31, 35, 36, 37, 38, 41, 42)
31-40	2 (9,19)
≥40	Not reported
Marital statues	
Married	14 (9, 13, 21, 22, 23, 25, 26, 27, 31, 33, 35, 38, 39, 41, 43)
Single	11 (8, 12, 14, 16, 17, 29, 34, 40, 42, 44)
Number of children	
0	6 (12, 14, 17, 18, 21, 44)
1-2	11 (9, 13, 16, 19, 25, 31, 34, 35, 37, 41, 43)
≥3	4 (22, 23, 38, 39)
Educational level	
Illiterate	2 (13, 23)
Lower than high school	16 (14, 15, 16, 17, 18, 19, 22, 27, 29, 37, 38, 39, 40, 41, 43, 44)
High school and above	8 (8,9,21, 25, 26, 31, 35,42)
Employment status	
Unemployed	16 (8, 12, 13, 14,17, 23, 25, 26, 27, 29, 35, 37, 38, 40, 43, 44)
Employed	7 (9, 18, 21, 31, 39, 41,42)
Socioeconomic status	
Low	10 (7,13, 16, 17, 21, 27, 38, 39, 40, 42)
Moderate	3 (33, 35, 37)

**Table 4:** Types of the illegal abortion service providers in the included studies within the systematic review of the research evidence on cross-country features of illegal abortions

**Service providers**	**No. of reporting studies**
Patient	5 (12, 13, 25, 31, 41)
Midwife	5 (13, 25, 34, 36, 43)
Friend/relative	2 (13, 37)
Traditional healer	2 (29, 37)
Gynecologist	2 (24, 35)
General practitioner	1 (12 )

**Table 5:** Stated reasons to request an illegal abortion in the included studies within the systematic review of the research evidence on cross-country features of illegal abortions

**Stated reasons**	**No. of reporting studies**
Having enough number(s) of child(ren)	10 (9, 13, 15, 23, 32, 35, 36, 37, 41, 43)
Proper spacing between deliveries	3 (19, 20, 22)
To continue education	3 (12 ,14, 29)
Fear of public or parents misjudgment	2 (12, 25)
Poor economical status	1 (18)
Being single	1 (12)
Not being able to afford a baby	1 (30)
Relationship problems with partner	1 (7)

**Table 6:** Reported illegal abortion places in the included studies within the systematic review of the research evidence on cross-country features of illegal abortions

**Abortion places**	** No. of reporting studies**
Private hospitals	4 (20, 23, 28, 33)
Private house	3 (25, 34, 37)
Private office	3 (20, 28, 37)
Public hospitals	2 (14, 34)

## References

[1] Admasie Gelaye A, Nigussie Taye K, Mekonen T (2014). Magnitude and risk factors of abortion among regular female students in Wolaita Sodo University, Ethiopia. BMC Womens Health.

[2] Abera GB, Berhanu B, Kahsay AB, Gebru HB, Aregay A (2012). Assessment of determinants of induced abortion among child bearing age women attending maternal and child health clinic in Mekelle town, Tigray, Ethiopia: a cross sectional study. Int J Pharm Sci Res.

[3] Faúndes A (2016). What can we do as gynecologists/obstetricians to reduce unsafe abortion and its consequences? The Uruguayan response. Int J Gynaecol Obstet.

[4] Cameron ST, Riddell J, Brown A, Thomson A, Melville C, Flett G (2016). Characteristics of women who present for abortion towards the end of the mid-trimester in Scotland: national audit 2013-2014. Eur J Contracept Reprod Health Care.

[5] World Health Organization. Unsafe abortion: global and regional estimates of the incidence of unsafe abortion and associated mortality in 2008. 6th ed. Geneva, Switzerland: WHO; 2011.

[6] Gerdts C, Vohra D, Ahern J (2013). Measuring unsafe abortion-related mortality: a systematic review of the existing methods. PLoS One.

[7] Osur J, Orago A, Mwanzo I, Bukusi E (2015). Social networks and decision making for clandestine unsafe abortions: evidence from Kenya. Afr J Reprod Health.

[8] Ilboudo PG, Somda SM, Sundby J (2014). Key determinants of induced abortion in women seeking postabortion care in hospital facilities in Ouagadougou, Burkina Faso. Int J Womens Health.

[9] Erfani A (2011). Induced Abortion in Tehran, Iran: estimated rates and correlates. Int Perspect Sex Reprod Health.

[10] The PLOS Medicine Editors (2014). Observational studies: getting clear about transparency. PLoS Med.

[11] Tong A, Sainsbury P, Craig J (2007). Consolidated criteria for reporting qualitative research (COREQ): a 32-item checklist for interviews and focus groups. Int J Qual Health Care.

[12] Koster-Oyekan W (1998). Why resort to illegal abortion in Zambia? Findings of a community-based study in Western Province. Soc Sci Med.

[13] Ahmed S, Islam A, Khanum PA, Barkat EK (1999). Induced abortion: What’s happening in rural Bangladesh. Reprod Health Matters.

[14] Rasch V, Muhammad H, Urassa E, Bergström S (2000). The problem of illegally induced abortion: results from a hospital-based study conducted at district level in Dar es Salaam. Trop Med Int Health.

[15] Uygur D, Erkaya S (2001). Reasons why women have induced abortions in a developing country. Eur J Obstet Gynecol Reprod Biol.

[16] Mogilevkina I, Hellberg D, Nordstrom ML, Odlind V (2000). Factors associated with pregnancy termination in Ukrainian women. Acta Obstet Gynecol Scand.

[17] Rasch V, Wielandt H, Knudsen LB (2002). Living conditions, contraceptive use and the choice of induced abortion among pregnant women in Denmark. Scand J Public Health.

[18] Larsson M, Aneblom G, Odlind V, Tyden T (2002). Reasons for pregnancy termination, contraceptive habits and contraceptive failure among Swedish women requesting an early pregnancy termination. Acta Obstet Gynecol Scand.

[19] Ban DJ, Kim J, De Silva WI (2002). Induced abortion in Sri Lanka: who goes to providers for pregnancy termination?. J Biosoc Sci.

[20] Ganatra B, Hirve S (2002). Induced abortions among adolescent women in rural Maharashtra, India. Reprod Health Matters.

[21] Sihvo S, Bajos N, Ducot B, Kaminski M (2003). Women’s life cycle and abortion decision in unintended pregnancies. J Epidemiol Community Health.

[22] Perera J, de Silva T, Gange H (2004). Knowledge, behaviour and attitudes on induced abortion and family planning among Sri Lankan women seeking termination of pregnancy. Ceylon Med J.

[23] Bozkurt AI, Oezcirpici B, Ozgur S, Sahinoz S, Sahinoz T, Saka G (2004). Induced abortion and effecting factors of ever married women in the Southeast Anatolian Project Region, Turkey: a cross sectional study. BMC Public Health.

[24] Senbeto E, Alene GD, Abesno N, Yeneneh H (2005). Prevalence and associated risk factoprs of Induced Abortion in Northwet Ethiopia. Ethiopian Journal of Health Development.

[25] Adanu RM, Ntumy MN, Tweneboah E (2005). Profile of women with abortion complications in Ghana. Trop Doct.

[26] Nojomi M, Akbarian A, Ashory-Moghadam S (2006). Burden of abortion: induced and spontaneous. Arch Iran Med.

[27] Lara D, Garcia SG, Ellertson C, Camlin C, Suarez J (2006). The measure of induced abortion levels in Mexico using random response technique. Sociol Methods Res.

[28] Maral I, Durukan E, Albyrak S, Öztimur N, Biri A, Bumin MA (2007). Induced abortion frequency in Ankara, Turkey, before and after the legal regulation of induced abortion. Eur J Contracept Reprod Health Care.

[29] Dahlbäck E, Maimbolwa M, Kasonka L, Bergström S, Ransjö-Arvidson AB (2007). Unsafe induced abortions among adolescent girls in Lusaka. Health Care Women Int.

[30] Hess RF (2007). Women’s stories of abortion in southern Gabon, Africa. J Transcult Nurs.

[31] Tsakiridu DO, Vidal AF, Valdés FV, Junquera Llaneza ML, Varela Uría JA, Cuesta Rodríguez M (2008). Factors associated with induced abortion in women prostitutes in Asturias (Spain). PloS One.

[32] Rahim N, Ara A (2008). Reasons due to which, women resort to illegally induced abortions. J Postgrad Med Inst.

[33] Dibaei A, Saadati N. A survey of prevalence, demograohy characteristics, causes and side effects of abortion in clients referred to hospitals of Ahwaz University in 2004. Jundishapur Scientific Medical Journal 2008;7(1):12-22. [Persian].

[34] Rasch V, Kipingili R (2009). Unsafe abortion in urban and rural Tanzania: method, provider and consequences. Trop Med Int Health.

[35] Motavalli R, Alizadeh L, Namadi Vosoughi M, Shahbazzadegan S. Evaluation of the prevalence, reasons and consequences of induced abortion in women of Ardabil in 2011. Journal of Ardabil University of Medical Sciences 2012;12(4):384-91. [Persian].

[36] Veisi F, Zangene M (2012). The causes of illegal abortions and their methods in outpatient clinics of Kermanshah University of Medical Sciences. Sci J Forensic Med.

[37] Ranji A (2012). Induced abortion in Iran: prevalence, reasons, and consequences. J Midwifery Womens Health.

[38] Nur N (2012). Socioeconomic disparities among ever-married Turkish women who had unintended pregnancies and abortions in a middle Anatolian city. Women Health.

[39] Souza MG, Fusco CL, Andreoni SA, de Souza e Silva
 R
 (2014). Prevalence and sociodemographic characteristics of women with induced abortion in a population sample of São Paulo, Brazil. Rev Bras Epidemiol.

[40] Fusco CL, de Souza e Silva R, Andreoni S (2012). Unsafe abortion: social determinants and health inequities in a vulnerable population in Sao Paulo, Brazil. Cad Saude Publica.

[41] Rocca CH, Puri M, Dulal B, Bajracharya L, Harper CC, Blum M (2013). Unsafe abortion after legalisation in Nepal: a cross-sectional study of women presenting to hospitals. BJOG.

[42] Motaghi Z, Keramat A, Shariati M, Yunesian M (2013). Triangular assessment of the etiology of induced abortion in Iran (a qualitative study). Iran Red Crescent Med J.

[43] Awoyemi BO, Novignon J (2014). Demand for abortion and post abortion care in Ibadan, Nigeria. Health Econ Rev.

[44] Klutsey EE, Ankomah A (2014). Factors associated with induced abortion at selected hospitals in the Volta Region, Ghana. Int J Womens Health.

